# Pediatric hospital visits for unintentional drowning in bathtubs in Central Texas, USA

**DOI:** 10.1186/s40621-025-00597-7

**Published:** 2025-07-07

**Authors:** Molly B. Johnson, Barbara D. Cosart, Stewart R. Williams, Brent M. Troy, Karla A. Lawson

**Affiliations:** 1 Trauma and Injury Research Center, Dell Children’s Medical Center, 4900 Mueller Blvd, Austin, TX 78723 USA; 2 Kinesiology Department, University of the Incarnate Word, San Antonio, TX 78209 USA; 3 Trauma Services, Dell Children’s Medical Center, 4900 Mueller Blvd, Austin, TX 78723 USA; 4 Department of Pediatrics, Dell Medical School, University of Texas at Austin, Austin, TX 78712 USA; 5 Department of Surgery and Perioperative Care, Dell Medical School, University of Texas at Austin, Austin, TX 78712 USA

**Keywords:** Drowning, Supervision, Public Health, Hospital Care, Pediatric

## Abstract

**Background:**

In the USA, drowning is a leading cause of death for children and the leading cause of death for children 1–4 years old. Bathtubs pose the highest risk of drowning for infants. The aim of this study is to determine factors that increase the risk of drowning in a bathtub for children.

**Methods:**

This retrospective, cross-sectional study used data retrieved from a hospital-based registry of drowning patients that includes information manually abstracted from patient medical records. This study describes patient characteristics and incident scenarios for children aged 0–17 years who sought care at one children’s hospital for unintentional drowning in a bathtub over a ten-year period, 2014- 2023. Chi-square analysis was used to assess associations between whether a supervising caregiver was present during the incident and the likelihood of hospital admission or the likelihood of poor outcome.

**Results:**

There were 50 patients 0–9 years old treated for unintentional drowning in a bathtub over the 10-year period. The majority of patients were female (62%), White (86%), or not Hispanic (53%). Most of the patients (84%) were under 2 years old and the majority (56%) were under 1 year old. For most of the patients 2–9 years old, the drowning incident was likely seizure-related. In 91% of the incidents, a caregiver was intending to supervise the child in or around the bath, yet in only 24% of the incidents was the caregiver engaged in supervising the child. The most common reasons for the lapse in supervision was that the caregiver was retrieving a towel and/or clothes (39%) or caring for other children (20%). Chi-square analysis showed that children who were admitted to the hospital for further care were more likely to have no adult caregiver present than those who were discharged after being treated in the Emergency Department only.

**Conclusions:**

Findings indicate that lapses in supervision are a common cause of bathtub drowning for young children and are associated with the need for higher levels of care. Additionally, results highlight the need for drowning prevention messaging emphasizing gathering all bath supplies before starting a bath and avoiding distractions, such as caring for other children.

## Background

Drowning is a major public health issue worldwide [1]. In the United States (USA), drowning is the leading cause of death for children 1–4 years old [2]. Drowning is fatal in 13% of pediatric drowning incidents [3]. Many children suffer from submersion injuries following a non-fatal drowning incident [2, 4]. In addition to the many children who experience a fatal or non-fatal drowning incident, many more children may be evaluated in hospitals after being rescued from a submersion incident.

In Texas and across the USA, the setting in which most drownings occur varies depending on the age of the victim. Adults, teenagers, and older children most often drown in natural water, such as oceans and lakes; children 1–4 years old most often drown in swimming pools; and infants under 1 year old most often drown in bathtubs [5, 6]. For all age groups combined, bathtubs account for 10% of drowning fatalities in Texas [5].

Infants and young children are at risk of drowning because they may be too young to have reached developmental milestones for movement control and cognitive problem-solving abilities that would enable them to lift their head, roll over, or pull themselves up if they become submerged under water [7]. Without the ability to bring their airway back above water, infants will not be able to adequately oxygenate their lungs and may aspirate liquid into their lungs; they could be in cardiac arrest within five minutes [8]. Even brief lapses in caregiver supervision during bathtime can put infants and young children at risk of severe drowning injury or death [9].

Prior research suggests that lapses in supervision are common in bathtub drowning incidents [10, 11]. Yet, many parents may not be aware of the importance of bathtub supervision for infants and young children. Caregivers of small children may not have adequate knowledge about the high risk of drowning for children, the seriousness of drowning injury, or tactics to improve water safety [12, 13]. In fact, studies have shown that some parents think they can briefly leave their infant without adult supervision when bathing [14–16].

The aim of this study is to investigate the epidemiology of pediatric patients who sought care at a hospital for a submersion or drowning incident involving a bathtub. Additionally, we aimed to identify common circumstances leading to bathtub drowning, including lapses in supervision, that could be useful for drowning prevention initiatives.

## Methods

This retrospective, cross-sectional cohort study assessed characteristics of patients, incident scenarios, and outcomes for children treated for unintentional drowning in a bathtub at one large urban pediatric hospital, over the 10-year period between January 1, 2014 and December 31, 2023. Data were retrieved from the hospital Submersion and Drowning Registry, a registry of hospital submersion patients maintained by research staff and populated using medical chart review. Two authors oversee the medical record abstraction for all patients in the registry, reconciling differences through discussion and documentation. The submersion registry data are collected and managed using REDCap (Research Electronic Data Capture) electronic data capture tools [17, 18]. The submersion registry includes patients identified as experiencing a submersion or drowning incident or a water rescue based on initial encounter ICD-9 codes (E830.*, E832.*, E910.*, E954.*, E964.*, E984.*, E995.4*, 994.1*) or ICD-10 codes (ending in A and having a 1 in the sixth digit indicating “causing drowning and submersion” in the range W16.011-W16.331 and W16.511-W16.831 or beginning with W16.41, W16.91, W22.041, W65-W74, X71, X92, Y21, or T75.1). Additionally, cases may have been included in the registry based on a search of the chief complaint of the hospital presentation using the *drown* string pattern, with * representing a wildcard character used to stand in for unknown characters for code and keyword searches. Patients are excluded from the registry if it is determined that they were not submerged or immersed in liquid based on medical chart review. The registry includes patients initially or solely treated in the emergency department (ED) and patients admitted to the hospital or directly admitted after transfer from another hospital. Incidents are only characterized as intentional drowning when another person actively caused the submersion, e.g. pushed the child under water (and in one instance, a news report of homicide admission). This may not be the same as determinations by medical examiners, who may code lapses in supervision as homicide, particularly for bathtub drownings involving very young children.

Descriptive data are presented for age, sex, race, ethnicity, year, month, and time of day. Incident scenario factors include data on whether the water time was planned, the activity the child was involved in at the time of the incident, whether a caregiver was present, whether only another child or teen was present, whether the caregiver was intending to supervise the child around water, and what activity the caregiver was involved in at the time of the incident (supervising/bath-related tasks/caring for other children/chores/on phone or texting/other). In some instances, a caregiver was present, but their activity was not coded as supervising because they were engaged in another activity, such as turning away to get a towel or on the phone. Additionally data is presented on whether the child was conscious when removed from the water, whether a Cardiopulmonary Resuscitation (CPR)-based activity was performed immediately after removing the child from the water (includes any use of rescue breathing, compressions, abdominal thrusts, pats on the back, or other CPR-like actions used with the intention of promoting breathing and/or circulation), and whether CPR was performed by a medical professional after arrival of Emergency Medical Services (EMS) or at the hospital. Data are also presented on hospital admission (treated in emergency department only/admitted to hospital) and outcome (death/morbidity—continued medical specialist or hospital care related to the drowning needed at discharge/no morbidity). Unknown data are excluded from percentages.

Fisher’s exact test was used to assess if there is an association between whether a caregiver was present and outcome (no morbidity/poor outcome—morbidity or death). Chi square analysis was used to assess if there is an association between whether a caregiver was present and hospital admission. Patients who died in the ED were excluded in this analysis.

## Results

### Demographic characteristics

Of the 457 pediatric patients treated for unintentional drowning or submersion at the hospital over the 10-year period, 50 (11%) occurred in a bathtub. Patients treated for drowning in a bathtub ranged in age from 0 to 9 years old. The majority of the patients were infants < 1 year old (56%) and most patients were < 2 years old (84%). The majority of patients were female (62%), White (86%), or not Hispanic (53%) (Table 1).

**Table 1:** Demographic characteristics of unintentional pediatric bathtub submersion patients

Variable	Category	n	Percentage
Age	< 1 year	28	56
	1 year	14	28
	2 years	2	4
	3 years	2	4
	4 years	0	0
	5 years	2	4
	6 years	0	0
	7 years	1	2
	8 years	0	0
	9 years	1	2
Sex	Female	31	62
	Male	19	38
Race	Black or African American	4	8
	White	42	86
	Other Race	3	6
Ethnicity	Hispanic	20	47
	Not Hispanic	23	53
Total patients		50	100

Note: Does not include data when race or ethnicity was not known

## Temporal characteristics

**Fig. 1:**
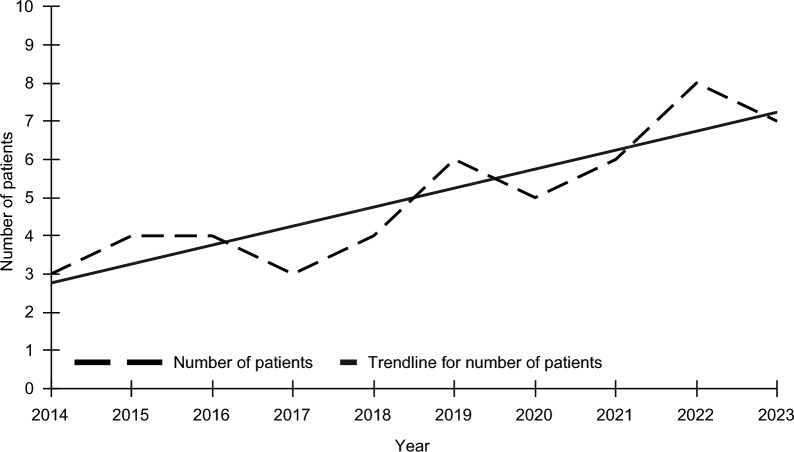
Number of patients treated for unintentional drowning in a bathtub in Central Texas by year, 2014–2023

The incidents occurred during every month, with the most submersions occurring in September (14%) and the fewest occurring in August (4%). The bathtub submersion incidents were most likely to occur late afternoon or evening, 4 pm-7:59 pm (48%) or 8 pm-11:59 pm (29%), with fewer occurring in the morning, 8am-11:59am (15%), or afternoon, 12 pm—3:59 pm (8%). The fewest bathtub drowning incidents were treated at the hospital in 2014 (6%) and the most in 2023 (14%) (Fig. 1).

## Precipitating factors

The bathtub submersion was precipitated by a suspected seizure in 12% (n = 6) of the patients, including 3/4 of patients more than 1 year old and all of the patients 7–9 years old.

## Incident scenarios and supervision

Of the bathtub submersion incidents, 96% (n = 48) were during a time when the parent planned to have the child in or around water and 4% (n = 2) were not during planned water time. The child was bathing during 94% (n = 47) of the incidents, with 2% (n = 1) of incidents occurring when a sibling was bathing, and 4% (n = 2) occurring when the child entered the bathroom without the caregivers’ knowledge. There was an adult caregiver present (e.g. in the bathroom) in 35% (n = 17) of incidents and no adult present in 65% (n = 31) of incidents. Only another child or teen was present in 31% (n = 15) of the incidents.

In 91% (n = 42) of the incidents, a caregiver was intending to be present and supervise the child in or around a bath. Despite the fact that a caregiver intended to supervise, an adult caregiver was only engaged in supervising (and no other activities) in 24% of the incidents, with a lapse in supervision by the caregiver in 76% of the incidents. Bath-related tasks were the most common caregiver activity during the lapse in supervision (39%), with all of the bath-related tasks involving retrieving a towel and/or clothes (Table 2).

**Table 2:** Caregiver activity during unintentional pediatric bathtub submersion incident

Caregiver activity	n	Percentage
Bath-related tasks	16	39
Supervising	10	24
Caring for other children	8	20
Chores (unrelated to bath time)	2	5
On phone or texting	2	5
Other: watching TV, using restroom, answering door	3	7
Total	41	100

Note: does not include data when caregiver activity was not known

## Outcomes

The patient was not breathing when removed from the water in 46% (n = 22) of the incidents and was not conscious in 42% (n = 21) of the incidents. A CPR-based rescue activity was performed immediately after removing the child from the water in 54% (n = 27) of the incidents. CPR was performed by a medical professional after arrival of EMS or at the hospital in 12% (n = 6) of the incidents.

Thirty-eight percent (n = 19) of the patients were treated in the ED only; 60% (n = 30) of the patients were admitted to the hospital. Two percent (n = 1) of the patients expired before being admitted to the hospital. Fourteen percent (n = 7) of the patients were treated in the intensive care unit (ICU). The outcome of the incident was no morbidity for 88% (n = 44) of the patients, morbidity for 8% (n = 4) of the patients, and death for 4% (n = 2) of the patients.

## Outcome and supervision

Pearson’s chi-square test showed an association between whether a caregiver was present at the time of the incident and hospital admission (X^2^ = 9.3935, *P* = 0.002), with children admitted to the hospital more likely to have no adult caregiver present at the time of the incident than those treated in the ED only (Table 3). A Fisher’s exact test did not show a significant association between whether a caregiver was present at the time of the incident and the outcome (*P* = 0.077).

**Table 3:** Association of whether a caregiver was present and hospital admission for unintentional pediatric bathtub submersion patients

	Hospital admission	Treated in emergency department only	Total
No adult caregiver present	80%	35%	64%
Adult caregiver present in same space	20%	65%	36%
Total	100%	100%	100%

## Discussion

Results from this study indicate that although caregivers usually intend to supervise their young children during bathtime, lapses in supervision are a main driver of pediatric bathtub drowning incidents. Additionally, our data highlight that children requiring a higher degree of care (hospital admission) were more likely to have no caregiver present at the time of the incident compared to those needing less intensive treatment (care in the ED only). Prior research has shown that indoor household duties are the most common distraction during lapses in supervision associated with drowning, with gathering clothing, towels, or shampoo as the common reasons for lapses in supervision during baths [19, 20]. This study highlights the single most common caregiver activity during bathtub drowning in our cohort: more than one third of caregivers were retrieving a bath towel and/or clothes when their supervision lapsed. Bath drowning prevention messaging should encourage parents and caregivers to gather towels and clothes prior to placing an infant in a bath.

Our results showing that 84% of bathtub drowning patients were < 2 years old suggest that infants and toddlers should have supervision while bathing. Caregivers should be advised to never leave an infant in a bathtub without an adult present for any reason. The American Academy of Pediatrics suggests that adult supervision around water should be close, constant, and attentive [21]. Our results indicate that children 2–5 years old can also be at risk of drowning in a bathtub and should be monitored while bathing. Active play during bathtime and breath holding can be hazardous for children who can slip or accidentally inhale water, so the presence of an adult provides the best injury prevention.

Our data show that it is common to leave infants and young children alone with other children or teens, but caregivers should be advised that children cannot act as substitutes for adult supervision and, in some cases, children can increase the risk of infant drowning (e.g. by closing the bathtub drain so that the tub fills over the infant’s head). Prior research shows it is common for children to drown in bathtubs while co-bathing with a sibling and that children who drown in bathtubs often have older siblings [10, 22, 23].

Additionally, children with a history of seizures are at risk of drowning while bathing and steps should be taken to monitor children with a history of seizures while in a bathtub. Research from Australia shows that medical conditions with seizures are the most prevalent condition associated with drowning for children [24]. Prior research from Canada indicates that people with epilepsy are at 6.3 times greater risk of drowning than the general population and that the majority of drownings related to seizures occur in a bathtub [25, 26]. Parents of children with seizure disorders should be educated about increased drowning risk for their child and provided with drowning prevention tips during bathing and other water activities.

Prior research indicates a prevalence of male drownings when all settings and ages are combined, with potentially large disparities depending on the scenario (e.g. 90% of drowning fatalities are male for 15–19-year-olds in Texas) [5]. However, this disparity by sex is not reflected in our pediatric bathtub drowning data. Our data show a slightly higher proportion of female patients (62%). It is likely a larger sample would show a relatively equal proportion of male and female pediatric bathtub drowning patients, as is seen in prior research on bathtub drowning [9].

Drowning incidents that occur in home pools often occur during non-swim times, when a child may access the pool without permission or knowledge of the caregiver [27]. Bathtub drowning incidents, however, mostly happen during planned bathtime (96% in this study), with the incident happening due to a lapse in caregiver supervision. However, care should be taken to protect against unplanned bath access, particularly if the bathtub is intentionally filled, such as during a water shortage. About one third of children 10–18 months old are able to climb into a bathtub [28]. It is suggested that during emergency situations when water utilities are shut off, recommendations to fill bathtubs with water should be qualified with brief acknowledgements of the risk to infants and toddlers and the importance of preventing access to a bathtub with any amount of water in it for families with small children.

Drowning is preventable. Research generally shows improved water safety practices following a variety of water safety interventions ranging from social media posts to parent classes [29–32]. For bath safety, interventions provided to new parents or during prenatal check-ups may be useful. One study showed that parents who received tailored safety advice from pediatricians based on safety behavior assessments decreased their unsafe bathing practices compared to a control group who received generic safety information [32]. These findings indicate that prenatal and pediatrician visits should stress the importance of supervision while bathing young children. Further research is needed to evaluate additional injury prevention tactics for improving bath safety.

This study only analyzed patients who sought care at a hospital for a submersion or drowning incident. Over 1/3 of the patients were discharged from the ED after being evaluated or treated. Because infants cannot talk and young children may not be able to communicate their experience or symptoms clearly, parents may choose to bring young children in for evaluation following a submersion incident to be on the safe side. The World Health Organization (WHO) defines drowning as “the process of experiencing respiratory impairment from submersion/immersion in liquid” [33]. It is likely that many of these patients would not be defined as drowning by the WHO. Instead, they might be considered rescues and not included in data on fatal and non-fatal drowning. The authors of this study believe there is value in understanding the scenarios that led to the submersion incidents, whether or not the patient subsequently suffered respiratory symptoms, and so we chose to include all patients who sought care at the hospital for drowning or submersion in a bathtub.

This study assessed data for only one hospital, in one region of the USA Therefore, a limitation of the study is that the results may not be generalizable to other or larger populations. This study also only studied the pediatric population. Drowning most often occurs in bathtubs for infants < 1 year old and in other settings for older children and adults; however, some data suggest that bathtubs may again become a common drowning setting for adults over age 75 or 85 [5, 34, 35]. Future research on drowning should include assessment of drowning settings for older adults, when possible.

## Conclusions

The overwhelming majority of patients treated for drowning had no adult caregiver present in this study, which indicates the critical nature of caregiver supervision as a preventative measure for pediatric bathtub drowning. Based on our data, we recommend close, constant, and attentive adult supervision during baths for young children. Additionally, we recommend monitoring for older children, particularly those with a history of seizures. We found that caregivers frequently stopped supervising their child in order to retrieve a bath towel and/or clothes. Our results suggest that these recommendations be prioritized in bath safety messaging: 1) to gather bathing supplies prior to running water for a child’s bath, 2) to take an infant out of the bath if leaving for any reason, and 3) to turn off phones and avoid distractions or caring for other children when bathing a child.

## Data Availability

Data can be made available through written request to the corresponding author.

## Abbreviations

CPR: Cardiopulmonary Resuscitation
ED: Emergency department
EMS: Emergency Medical Services
ICU: Intensive Care Unit
REDCap: Research Electronic Data Capture
USA: Unites States of America
WHO: World Health Organization
